# From Spatial Heterogeneity to Real-Time Monitoring: Liquid Biopsy for Genomic Profiling and MRD Assessment in Multiple Myeloma

**DOI:** 10.3390/cancers18091439

**Published:** 2026-04-30

**Authors:** Fizza Rasheed, Yafeng Ma, Therese M. Becker, Tara L. Roberts, Silvia Ling

**Affiliations:** 1 School of Medicine, Western Sydney University, Campbelltown, NSW 2560, Australia; yafeng.ma@unsw.edu.au (Y.M.); therese.becker@inghaminstitute.org.au (T.M.B.); tara.roberts@westernsydney.edu.au (T.L.R.); silvia.ling@health.nsw.gov.au (S.L.); 2 Ingham Institute for Applied Medical Research, 1 Campbell Street, Liverpool, NSW 2170, Australia; 3 South Western Sydney Clinical School, University of New South Wales, Liverpool, NSW 2170, Australia; 4 Department of Haematology, NSW Health Pathology, Liverpool Hospital, Liverpool, NSW 2170, Australia

**Keywords:** liquid biopsy, multiple myeloma, mutation profiling, cell-free DNA, circulating tumor cells, minimal residual disease, mass spectrometry, personalized medicine

## Abstract

Multiple myeloma (MM) is a malignancy of plasma cells that is characterized by a complex and spatially heterogeneous genomic landscape. Despite this complexity, clinical monitoring remains largely dependent on localized bone marrow (BM) assessments. This dependence creates a significant diagnostic gap, as the primary monitoring tools fail to account for the spatial and temporal heterogeneity that drives tumor relapse. Liquid biopsy can serve as an adjunctive approach in assessing the pan-clonal landscape in MM through the molecular profiling of circulating tumor DNA (ctDNA) and circulating tumor cells (CTCs). In this review, we examine the clinical utility of liquid biopsy components in capturing mutational profiles, clonal evolution, treatment resistance mechanisms, and minimal residual disease (MRD), including early detection of relapse and extramedullary progression. We will further discuss current limitations, including variability in assay sensitivity, lack of standardization, and the need for prospective validation.

## Simple Summary

Current routine management of multiple myeloma patients involves invasive bone marrow biopsies, which, due to common spatial disease heterogeneity, limit the detection of genetic biomarkers for patient care. Non-invasive liquid biopsies are emerging as an attractive adjunct for capturing the heterogeneous molecular landscape of multiple myeloma. Liquid biopsies enable dynamic examination of cell-free DNA and circulating tumor cells. Due to its minimal invasiveness, liquid biopsy has the potential to provide accessible, repeated assessment of comprehensive genetic profiles, tumor burden, clonal evolution, and minimal residual disease in multiple myeloma. Mass spectrometry is highly sensitive in detecting serum paraprotein and, as a minimal residual disease tool, has a similar prognostic significance to bone marrow next-generation flow cytometry. Liquid biopsy is emerging as a key research and clinical assessment tool in multiple myeloma, which may enhance personalized, response-adapted therapy and ultimately improve patient outcomes.

## 1. Introduction

Multiple myeloma (MM) is a malignancy characterized by the clonal proliferation of plasma cells, which are frequently distributed in a patchy manner within the bone marrow (BM). As the malignancy progresses, it may become more confluent or spread systemically with extramedullary involvement [1,2]. There have been significant advances in therapeutics, including proteasome inhibitors, immunomodulatory drugs, monoclonal antibodies, bispecific antibodies, and chimeric antigen receptor (CAR T-cell) therapies, which have extended the survival of MM patients [3,4]. However, MM remains an incurable disease, with a 5-year survival of approximately 61–63% based on the longitudinal cohorts of 2015–2021 [5]. While median overall survival for standard-risk patients now often exceeds 8–10 years because of quadruplet induction therapies and autologous stem cell transplantation (ASCT), the prognosis remains heterogeneous and heavily dependent on molecular risk profiles [6].

**Figure 1:**
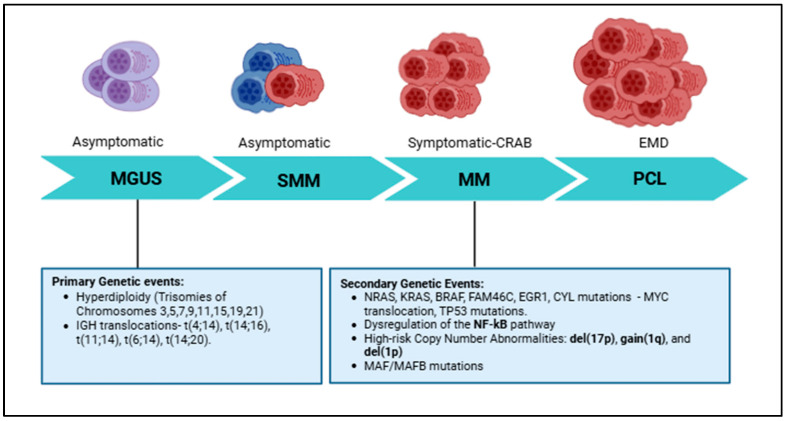
Stepwise disease progression and genomic evolution in plasma cell neoplasms. Schematic representation of disease evolution from monoclonal gammopathy of undetermined significance (MGUS) to smouldering multiple myeloma (SMM), multiple myeloma (MM), and primary plasma cell leukemia (PCL), representing extramedullary disease (EMD). MGUS and SMM are asymptomatic precursor states, whereas MM is characterized by end-organ damage defined by CRAB features (hypercalcemia, renal impairment, anemia, and bone lesions). Early disease stages are driven predominantly by primary genetic events, including hyperdiploidy (trisomies of chromosomes 3, 5, 7, 9, 11, 15, 19, and 21) and recurrent translocations involving the immunoglobulin heavy chain (IgH) locus, including t (4;14), t (14;16), t (11;14), t (6;14), and t (14;20). Disease progression is associated with the accumulation of secondary genetic alterations, including mutations in NRAS, KRAS, BRAF, FAM46C, EGR1, CYL, and MYC translocations; TP53 inactivation; dysregulation of the nuclear factor-κB (NF-κB) pathway; and high-risk copy-number abnormalities (CNAs), including del (17p), gain (1q), and del (1p), reflecting increasing clonal heterogeneity and biological aggressiveness.

The development of MM is a multi-step process that evolves from clinically defined precursor stages, beginning with Monoclonal Gammopathy of Undetermined Significance (MGUS), progressing to Smoldering Multiple Myeloma (SMM), and culminating in symptomatic MM [7]. This clinical and biological spectrum is driven by a complex and evolving genomic landscape, beginning with primary genetic events of hyperdiploidy and/or immunoglobulin heavy chain (IgH) translocations and advancing through secondary changes, including mutations in the RAS/RAF/MAPK pathway [8,9] (Figure 1).

The standard diagnosis and monitoring of MM, as recommended by the International Myeloma Working Group (IMWG), includes assessment of serum and urinary M-protein, serum-free light chains, skeletal imaging, BM aspirate and trephine, cytogenetic analysis by Fluorescence in Situ Hybridization (FISH), and mutational studies of the BM aspirate [10]. MM is frequently spatial and genetically heterogeneous both at diagnosis and at relapse/refractory stages. Multi-region sequencing studies revealed significant genomic differences between standard iliac crest biopsies and radiology-guided samples of focal lesions in approximately 75% of both newly diagnosed and relapsed/refractory multiple myeloma (RRMM) patients [11]. Advances in imaging techniques have been integrated into the standard staging of MM. However, the assessment of chromosomal copy-number abnormalities, translocations, and single-nucleotide variations in the routine clinical setting still relies on single-site BM biopsies. Notably, although such spatial heterogeneity is well documented in MM, its impact on clinical decision making remains limited, as the detection of site-specific subclonal mutations does not currently alter standard therapeutic strategies, which are primarily guided by overall disease burden and established risk factors. Deciphering and targeting these heterogeneous genomic changes remains an area of unmet clinical need [11].

As shown in various cancers, liquid biopsy leverages the analysis of circulating biomarkers such as circulating tumor DNA (ctDNA), circulating tumor cells (CTCs), extracellular vesicles (EVs), and tumor-educated platelets (TEPs), derived from peripheral blood and other biological fluids [12]. ctDNA and CTCs have been extensively studied in MM for their potential to capture genetic heterogeneity and disease complexity. ctDNA, which is a part of cell-free DNA (cfDNA), reveals somatic mutations, copy number variations, and chromosomal abnormalities, providing a real-time snapshot of the malignancy’s mutational landscape and being prognostically significant [13]. CTCs, on the other hand, are malignant plasma cells in the context of MM isolated from blood samples, often viable, which may serve as a source for genomic, transcriptomic, and proteomic analysis [14]. Both ctDNA and CTCs have been tested for their diagnostic, prognostic, and therapeutic significance in MM. EVs, a heterogeneous population of exosomes and microvesicles, and TEP are being explored as potential biomarkers for MM [15,16].

Assessment of minimal residual disease (MRD) has become a clinically meaningful endpoint in MM, given its strong association with progression-free survival (PFS) and overall survival (OS) [17]. Current MRD evaluation primarily relies on next-generation sequencing (NGS), or multiparameter/next-generation flow (NGF) cytometry performed on single-site BM aspirates. Recent studies demonstrate that post-treatment MRD detection using serum quantitative immunoprecipitation mass spectrometry is prognostically relevant and that conversion from MRD negativity to positivity is predictive of subsequent clinical progression [18]. Functional imaging modalities, including PET/CT and/or MRI, can identify residual disease at additional anatomical sites; however, their sensitivity and specificity, particularly in the setting of low disease burden, remain limited [19]. Accordingly, there is growing interest in evaluating non-invasive liquid biopsy approaches to complement existing MRD assessments and enable longitudinal monitoring, potentially mitigating the impact of clonal heterogeneity inherent to other techniques [20].

This review critically evaluates the emerging clinical utility of ctDNA and CTCs as diagnostic, prognostic, therapeutic, and MRD assessment tools in MM. It also highlights key gaps in the current evidence base and delineates the technical, analytical, and regulatory milestones required to facilitate the integration of liquid biopsy into routine clinical practice.

## 2. Cell-Free DNA: Leading Candidate

The concept of liquid biopsy in MM began with the discovery of nucleic acid fragments circulating in human blood, first described in 1948 by Mandel and Metais [21]. Released through biological processes such as apoptosis, necrosis, and active secretion from cells, cfDNA refers to DNA nucleic acid fragments of 160 to 180 base pairs [22,23]. While most cfDNA in healthy individuals comes from normal blood cell turnover, in cancer patients, a critical fraction consists of ctDNA [24]. Studies have established that ctDNA may serve as a molecular cancer fingerprint, as it can be distinguished from the background of normal cfDNA by tumor specific somatic mutations, chromosomal abnormalities, or copy number variations [25,26].

A key biological characteristic of cfDNA is its short half-life, estimated at 15 min to 2 h, as it is rapidly cleared from circulation by the liver and kidneys [27]. This rapid turnover means that ctDNA may provide a real-time snapshot of tumor burden and its clonal dynamics, often before clinical and radiographic relapse [28]. The clinical utility of ctDNA is already firmly established in solid tumor management, where it has transitioned from a research tool to a routine clinical application to guide treatment. For instance, in 2016, the US FDA approved the first blood-based ctDNA liquid biopsy for epidermal growth factor receptor (EGFR) genotyping in lung cancer patients [29]. Another illustrative example is metastatic colorectal cancer, a disease characterized by substantial clonal heterogeneity between primary and metastatic lesions. In this setting, ctDNA analysis is now routinely employed to detect KRAS mutations, which are predictive of resistance to anti-EGFR therapies such as cetuximab and panitumumab [30]. This approach enables effective patient stratification without the need for repeated invasive tissue biopsies [31].

### 2.1. Detection Methods of ctDNA in MM

In MM, ctDNA has been proposed to identify high-risk CNAs such as amp (1q), del (17p), and del (13q) [32]. This is primarily achieved through low-pass whole-genome sequencing (LPWGS) or targeted hybridization capture, which normalizes DNA fragment coverage across the genome to enable the detection of copy number imbalances. Deviations are inferred by fragment counting, whereby increased fragment density indicates chromosomal gains, while reduced signal reflects deletions [8]. The accuracy of CNA detection is further enhanced by estimating the tumor fraction (TF), the proportion of ctDNA relative to total cfDNA, which has been shown to carry independent prognostic significance in MM [27,32].

Complementing these broad genomic screens, targeted NGS allows for the simultaneous detection of single-nucleotide variations across recurrently mutated genes, such as KRAS, NRAS, and BRAF, providing a detailed map of clonal architecture [33]. Conversely, droplet digital PCR (ddPCR) offers high sensitivity and quantitative tracking of known mutations. By partitioning the sample into thousands of individual droplets, ddPCR enables the absolute quantification of rare mutant alleles even at extremely low TFs, making it a particularly well-suited approach for longitudinal MRD monitoring in peripheral blood [34,35].

### 2.2. ctDNA Concordance with Bone Marrow Tumor DNA

Multiple studies have reported varying levels of concordance between ctDNA and BM tumor profiles, as summarized in Table 1. Kis et al. utilized a hybrid capture liquid biopsy sequencing (LB-Seq) approach to analyze five key oncogenes (KRAS, NRAS, BRAF, EGFR, and PIK3CA) at a mean sequencing depth of >5000×. In a cohort of 53 patients, a concordance rate of 96% (49/51 mutations) was observed from 48 cfDNA specimens with matched BM specimens [36]. Such high concordance may imply that ctDNA merely replicates existing BM data without providing additional insights. The minor discordance was attributed to a very low variant allele frequency (VAF) of a KRAS p.G12D mutation (1.3%) in one BM sample, which was below the limit of detection in clinical-grade sequencing [36]. The LB-Seq platform demonstrated high analytical sensitivity, detecting ctDNA at VAFs as low as 0.25% while maintaining a specificity exceeding 98% [36]. These findings were further corroborated by Gerber et al., who employed Cancer Personalised Profiling by Deep Sequencing (CAPP-seq), an ultra-deep sequencing approach, with a depth of >1000×. In a cohort of 28 patients, this method achieved a detection sensitivity of 0.01% VAF and 72% concordance between paired BM and ctDNA samples, including patients with SMM and low-burden disease [37]. Lower concordance may reflect spatial and genetic heterogeneity rather than a methodological limitation.

The broader genomic landscape has been recently characterized by Martello et al. in a large prospective study of 162 newly diagnosed multiple myeloma (NDMM) patients. Utilizing ultra-low-pass whole-genome sequencing (ULP-WGS), the authors demonstrated that ctDNA reliably recapitulated the BM copy-number profile, with >75% of patients showing high concordance for major structural alterations. Notably, a ctDNA TF threshold of ≥12% (*p* value  =  0.0000782; AUC  =  0.585; Accuracy  =  0.66; Sensitivity  =  0.56; Specificity  =  0.73) was identified as an independent prognostic marker, which was associated with a higher total metabolic tumor volume on PET/CT. This finding highlights the capacity of ctDNA to act as a surrogate of disease burden, particularly in the RRMM setting [38]. Complementing this, Mithraprabhu et al. reported that while concordance remains high, a significant proportion of mutations are detected exclusively in plasma, particularly in RRMM patients (27.2%) compared with newly diagnosed cases (6.6%), highlighting the spatial and genetic heterogeneity of advanced disease [39].

This systemic view of disease heterogeneity was further supported by Coffey et al., who demonstrated that ctDNA captures subclones arising from distant or extramedullary sites not represented in a single BM sample. Through multisite sequencing of ctDNA, BM aspirate, and direct biopsies of extramedullary plasmacytomas in a cohort of 25 patients with RRMM, the authors showed that ctDNA can effectively capture subclonal mutational diversity across distinct tumor compartments [40].

In conclusion, the clinical utility of concordance lies not in its absolute magnitude but in its ability to balance faithful representation of BM disease with the detection of additional subclonal heterogeneity and should be interpreted within the appropriate clinical context.

**Table 1:** Analysis of sequencing methodologies and concordance rates for ctDNA samples vs. BM.

Sample Size	Technique	Mutational Frequency (ctDNA)	Concordance with BM/ Key Findings	Ref.
53 MM	LB-Seq hybrid capture; 5-gene panel	Actionable mutations: KRAS, NRAS, BRAF;	96% concordance, LB-Seq detects BM mutations with >98% specificity.	[36]
28 (2 MGUS, 5 SMM & 21 MM)	CAPP-seq ultra-deep targeted NGS; 14-gene panel	NRAS (25%), KRAS (14%), TP53, TRAF3 & FAM46C (11%)	72% (28/39) of mutations in BM PCs were correctly identified in cfDNA.	[37]
48 MM patients (15 ND, 33 RR)	OMD platform for KRAS, NRAS, BRAF, TP53	24.2% (*n* = 48) have mutations detected exclusively in plasma; 27.2% in RRMM vs. 6.6% in NDMM.	cfDNA analysis shows spatial heterogeneity and additional mutations.	[39]
162 NDMM MM patients	ULP-WGS and 18F-FDG PET/CT	ctDNA TF > 12% is defined as high-risk, correlating with systemic disease spreading.	cfDNA showed high overall concordance with BM clonal CNV profiles (46/62 patients with >75% concordance).	[38]
25 patients (RRMM)	Ultra-deep targeted sequencing (4100× depth for targeted ctDNA sequencing)	70 recurrently mutated genes in MM, which includes 63 known driver genes	Distinct mutations were detected in cfDNA that were not identified in matched BM samples, suggesting spatial heterogeneity.	[40]
261 RRMM (BM and ctDNA from Plasma)	Targeted sequencing (1085 genes in BMPC; 76 genes in ctDNA)	TP53 (21.6% in ctDNA and 12.9% in BM) and KRAS (10% in ctDNA; 21.5% in BM). 59.2% of TP53-mutated cases showed mutations exclusively in ctDNA.	ctDNA mutations were stronger prognostic predictors than BMPC mutations in RRMM patients.	[41]

MM: multiple myeloma; SMM: smouldering multiple myeloma; MGUS: monoclonal gammopathy of undetermined significance; RRMM: relapsed/refractory multiple myeloma; ND: newly diagnosed; RR: relapsed/refractory; cfDNA: circulating cell-free DNA; BM: bone marrow; BMPC: bone marrow plasma cell; PCs: plasma cells; NGS: next-generation sequencing; ULP-WGS: ultra-low-pass whole-genome sequencing; WGS: whole-genome sequencing; LB-Seq: liquid biopsy sequencing; CAPP-seq: cancer Personalized Profiling by Deep Sequencing; OMD: Oncomine cfDNA Assay platform.

### 2.3. Cell-Free DNA, a Prognostic Marker

In MM, quantifiable cfDNA concentrations represent a promising indicator of disease burden, with levels significantly higher than those observed in both healthy populations and other neoplastic conditions [7,42]. Clinical data reveal a stark contrast in plasma cfDNA levels: healthy subjects maintain low concentrations (mean: 15.21 ng/mL; median: 14.37 ng/mL), whereas patients with MM display markedly higher levels (mean: 275.35 ng/mL; median: 139.0 ng/mL). This increase is driven by rapid turnover and a high apoptotic rate of malignant plasma cells within the BM [43]. These elevated concentrations correlate strongly with disease progression and clinical activity, showing a significant positive correlation between cfDNA levels and established biomarkers, such as M-protein levels (r = 0.52; *p* < 0.0001), lactate dehydrogenase levels (r = 0.48; *p* < 0.0001), and the percentage of BM plasma cell infiltration (r = 0.45; *p* < 0.01) [44].

Beyond total concentration, the TF in cfDNA has emerged as a critical driver of risk stratification across the disease spectrum. Manier et al. demonstrated in a cohort of 107 patients that TF in cfDNA correlates directly with clinical progression, showing a marked increase as patients transition from SMM to active and relapsed states. They established that a TF ≥ 10% in patients with active MM, in association with elevated cfDNA levels, is a potent predictor of shorter PFS and OS, reflecting systemic disease burden that may be underrepresented by single-site BM aspirates [45]. This prognostic association appears consistent across different analytical platforms.

The prognostic utility of ctDNA in RRMM is centered on its ability to identify high-risk mutations. In a large prospective cohort of 261 RRMM patients treated with ixazomib, lenalidomide, and dexamethasone, Kogure et al. demonstrated that ctDNA mutations in KRAS, TP53, and ATM; the presence of a t (11;14) rearrangement; a higher number of prior therapies (≥3 regimens); and elevated plasma DNA concentration were independently associated with shorter PFS. Among these variables, KRAS exhibited the strongest prognostic impact [41]. Leveraging these findings, a prognostic score incorporating the number of ctDNA mutations, more than three prior treatment lines, and elevated plasma DNA concentration was developed to stratify PFS in RRMM, with 2-year PFS rates of 57.3% for patients with no detectable mutations, 22.7% for those with one mutation, and 0% for those harboring two or more mutations [41]. Collectively, these data support the use of ctDNA in predicting prognostic stratification in RRMM.

Overall, ctDNA TF and mutational profiling provide a robust, minimally invasive means of prognostic stratification in MM, capturing systemic disease burden and high-risk biology beyond single-site BM assessment. Standardization of analytical pipelines, assay thresholds, and prospective clinical validation will be essential to enable routine integration of ctDNA-based biomarkers into response-adapted care pathways.

### 2.4. Diagnosis and Monitoring of Oligo-Secretory and Non-Secretory Myeloma

The majority of MM secretes measurable paraprotein in the serum and/or urine either as an intact immunoglobulin or free light chains, which serve as established surrogates of tumor burden and form the standard response assessment [46]. However, approximately 1–2% of MM are nonsecretory diseases or oligo-secretory, for whom disease monitoring relies primarily on imaging and repeated BM biopsies [47]. These approaches are associated with radiation exposure and procedural invasiveness, respectively, and may be particularly burdensome for older or frail patients. In this context, liquid biopsy represents an attractive alternative. Hosoya et al. showed that ctDNA levels correlated with disease burden in a cohort of 8 oligo/non-secretory MM patients [48]. Larger, dedicated studies are required to validate ctDNA-based monitoring of oligo-secretory and non-secretory MM.

## 3. Exosomes as Emerging Biomarkers in Multiple Myeloma

In the clinical landscape of MM, exosomes, a subtype of EVs measuring 30–150 nm, have emerged as a potential analyte for MM biomarkers. These vesicles are characterized by a lipid bilayer that protects a diverse molecular cargo, including proteins, microRNAs (miRNAs), and long non-coding RNAs (lncRNAs) [49]. Beyond mere cellular waste disposal, MM-derived exosomes actively contribute to pathogenesis by promoting angiogenesis via the delivery of vascular endothelial growth factor A and the long noncoding RNA MALAT1 to endothelial cells, while simultaneously inducing immunosuppression through inhibition of natural killer cell activity [50,51]. Furthermore, they stimulate osteoclastogenic signaling, accelerating bone resorption, a hallmark of MM-related morbidity [14].

Exosomal genetic components reflect the molecular characteristics of the tumor and are particularly well-suited for longitudinal monitoring [16]. The structural resilience of the exosomal lipid bilayer protects genetic cargo from enzymatic degradation by DNases in the circulation. In practice, exosomal DNA is often co-isolated during standard cfDNA extraction, as commonly used silica-column and magnetic-bead-based methods utilize lysis buffers that disrupt vesicular membranes, releasing intra-exosomal DNA into the total cell-free fraction [52]. This combined analyte may provide a more comprehensive representation of the genomic landscape, including the detection of subclonal TP53 and KRAS mutations, thereby enhancing analytical sensitivity [53].

Specific miRNA signatures, most notably let-7c-5p, miR-20a-5p, and the miR-17-92 cluster, have demonstrated high accuracy in discriminating between precursor stages such as MGUS and active disease while also serving as prognostic indicators for progression-free survival, but require validation in larger cohorts [54,55]. This molecular profiling also extends to multidrug resistance. The identification of an augmented CD138−/P-gp+/CD34+ exosome population in peripheral blood provides a non-invasive window into drug resistance and clonal evolution [56,57]. Exosomal miR-18a has been identified as a potential biomarker for bortezomib response, as its presence correlates with altered redox balance in recipient cells. However, current evidence remains primarily correlative, and further functional studies are required to establish a causal role in chemoresistance [58].

Despite these advances, several challenges remain. A major limitation to the clinical translation of exosome-based diagnostics is the lack of standardized, high-throughput isolation protocols, as current methods such as ultracentrifugation often yield a heterogeneous mixture of tumor-derived and non-malignant vesicles (e.g., from platelets and red blood cells) [59]. This background noise necessitates the development of more selective and scalable technologies, including magnetic nanozymes (C-IONPs) and plasmon-based biosensors for real-time profiling, which are not yet widely implemented in routine clinical laboratories [60]. Overall, while exosomes may represent a promising complementary component of liquid biopsy, further methodological standardization and prospective clinical validation are required to establish their role in routine MM management.

## 4. Circulating Tumor Cells: An Insight into Myeloma Cell Biology

The hallmark of malignant tumors is that tumor cells may detach, enter blood vessels, and disseminate systemically. In MM, CTCs represent a resilient, viable subpopulation of malignant plasma cells that have evolved to survive independently of the protective BM niche. While the majority of plasma cells remain strictly dependent on the BM microenvironment for survival signals, the egress of CTCs marks a critical transition toward niche-independence [61]. Their translocation into the peripheral blood and subsequent colonization to distant skeletal or extramedullary sites drives the spatial genomic heterogeneity that complicates therapeutic targeting and facilitates systemic disease evolution [62]. By isolating these migratory subpopulations, CTC-based assays offer a minimally invasive window into the pan-clonal tumor landscape. This is an approach particularly advantageous in mitigating the sampling biases inherent to single-site BM aspirates, which frequently fail to capture the multifocal nature of MM [12].

Phenotypic profiling of CTCs has revealed distinct molecular features underlying their egress from the BM [63]. Paiva et al. demonstrated that CTCs occupy a unique phenotypic niche, clustering independently of their paired BM tumor cells in multi-dimensional protein expression maps. This shift is characterized by downregulation of multiple integrins, cytokine receptors, and adhesion molecules, including CD11a, CD33, CD38, CD49e/d, CD56, CD81, CD117, and CD138, which weakens the physical tethering of malignant plasma cells to the BM stroma [64]. Transcriptional profiling by Vandyke et al. further revealed that CTCs are enriched for molecular hallmarks of inflammation, hypoxia, and epithelial–mesenchymal transition while exhibiting downregulation of cell-cycle genes, suggesting that a localized hypoxic and pro-inflammatory BM microenvironment may induce proliferative arrest and drive mobilization of tumor cells into the peripheral circulation as a survival mechanism [65,66]. Beyond their mechanistic release, CTCs mirror the mutational landscape of both medullary and extramedullary disease sites with high but incomplete concordance; whole-exome sequencing has confirmed up to 93% mutational concordance between CTCs and BM-derived tumor cells [45], with discordant mutations frequently representing subclonal diversity from non-sampled compartments.

### 4.1. Diagnostic Significance

Recent advances in CTC detection, including magnetic enrichment of CD138-positive cells, multiparameter flow cytometry, microfluidic devices, and NGF cytometry, have substantially improved detection sensitivity [67]. A landmark study employing NGF cytometry to analyze 550 matched BM and peripheral blood samples from NDMM patients demonstrated that CTCs, although present at low frequencies, were detectable in 90% of patients, with levels ranging from 0.0002% to 12.6%, and that higher CTC levels were associated with adverse clinical features, including a diffuse MRI pattern and significant phenotypic divergence between BM and peripheral blood clonal cells [68]. Critically, CTCs can be identified even at the earliest preclinical stages of the disease: using highly sensitive NGF cytometry, CTCs are detectable in approximately 59% of MGUS patients and in nearly all individuals with SMM or overt MM [69]. This early detectability positions CTC analysis as a promising diagnostic adjunct, capable of stratifying patients with precursor disease and informing the timing of therapeutic intervention prior to symptomatic progression. However, because CTCs represent only the migratory fraction, earlier stages of the disease may be studied with more impact via serum-based cfDNA. Since cfDNA is released by the broad population of plasma cells residing within the BM, it may provide a more comprehensive and predictive reflection of the total tumor burden and prognosis during these precursor phases.

The addition of CTC analysis to complementary liquid biopsy analytes, particularly cfDNA, further strengthens diagnostic accuracy. Only moderate concordance exists between CTC and cfDNA findings, suggesting they capture distinct and non-redundant aspects of disease biology and thus provide complementary insights into tumor burden and heterogeneity [62]. This synergy is vital; while CTCs track the aggressive, mobile clones, cfDNA serves as a surrogate for the sessile, niche-dependent marrow population. Furthermore, a threshold of ≥2% CTCs in the peripheral blood has been proposed to define a plasma cell leukemia (PCL)-like phenotype; Jelinek et al. demonstrated that more than 2% circulating tumor plasma cells identifies a PCL-like MM subset characterized by particularly aggressive biology and poor clinical outcomes, reinforcing the value of CTC quantification in the diagnostic and risk assessment framework [70].

### 4.2. Prognostic Significance

Quantitative CTC enumeration carries robust, independent prognostic value across the MM disease spectrum. In the FORTE trial, a large prospective multicenter study enrolling 401 NDMM patients, CTCs were detectable using second-generation multiparameter flow cytometry in 67% of patients at diagnosis, and a threshold of ≥0.07% was identified as the cutoff with the highest prognostic value for PFS and OS. In this trial, patients were prospectively randomized to carfilzomib–lenalidomide–dexamethasone (KRd)–ASCT (four KRd consolidation cycles), 12 KRd cycles, or carfilzomib–cyclophosphamide–dexamethasone (KCd)–ASCT (four KCd consolidation cycles) [71]. Patients with undetectable CTCs had exceptional PFS regardless of complete response or MRD status, while those with CTC ≥ 0.07% demonstrated significantly inferior PFS in multivariable analysis incorporating ISS stage, LDH levels, and cytogenetic abnormalities, confirming CTC burden as an independent prognostic variable.

The graded, dose-dependent relationship between CTC burden and outcome was further quantified in a recent analysis of patients treated with daratumumab, bortezomib, lenalidomide, and dexamethasone (D-VRd), where higher CTC levels were strongly correlated with worse PFS (HR 1.36 [95% CI 1.15–1.60]; *p* = 0.0004), independent of ISS, cytogenetics, LDH, and treatment group, with 4-year estimated PFS rates decreasing progressively across CTC strata: 93% for CTC < 0.001%, falling to 48% for CTC ≥ 1%. In this study, CTC was characterized by the EuroFlow 2-tube, 8-color panel [72]. A study by Cheng et al. in a cohort of 191 NDMM patients further established a CTC threshold of 0.038% as an independent prognostic marker for both PFS and OS, with patients above this level demonstrating substantially inferior survival outcomes [73]. Another major study by Garcés et al. using NGF cytometry in 374 NDMM patients showed that a CTC threshold of ≥0.01% significantly correlates with inferior PFS and can upstage patients within the R-ISS framework [13]. Furthermore, multiple studies have consistently validated the strong independent prognostic significance of CTC burden [74].

### 4.3. Comparison Between CTCs and cfDNA

CTCs and ctDNA represent the two most extensively characterized liquid biopsy analytes in MM, yet they provide fundamentally distinct and complementary windows into disease biology. Table 2 summarizes their key differences across methodology, genomic profiling capacity, clinical utility, and principal limitations. In brief, CTCs are intact, viable tumor cells that enable cellular-level interrogation, including transcriptomic and proteomic analysis, and offer a direct readout of tumor phenotype and functional behavior. In contrast, ctDNA consists of tumor-derived DNA fragments shed into the plasma, offering high-sensitivity genomic mutation tracking at scale and across a broad clonal landscape. A significant hurdle for ctDNA monitoring is the confounding presence of clonal hematopoiesis, which generates biological noise via age-related somatic mutations in genes such as DNMT3A, TET2, and ASXL1. Distinguishing these from myeloma-specific signals requires the routine use of paired white blood cell sequencing to bioinformatically filter hematopoietic variants and ensure high analytical specificity for MRD assessments [75].

The two analytes capture different compartments of the disease. CTC-based approaches uniquely capture the egress signature of migratory subclones and support real-time enumeration as an independent prognostic variable [16]. Conversely, ctDNA captures the genetic material of cells still tethered to the BM microenvironment; it may be more predictive of overall prognosis in early disease stages by reflecting the primary tumor’s evolution before systemic dissemination is widespread. While ctDNA achieves high concordance with clonal BM mutations (up to 96% in some series) and frequently detects plasma-exclusive mutations missed by single-site biopsies, the moderate concordance observed between these two modalities suggests they capture distinct, non-redundant aspects of tumor heterogeneity, a fact that serves as a compelling argument for their combined use.

### 4.4. CTCs and cfDNA for Comprehensive Genomic Profiling for MM

The genomic interrogation of CTCs by whole-exome sequencing and targeted NGS has generated compelling evidence that these approaches can recapitulate and, in some respects, exceed the cytogenetic resolution achievable by FISH, while simultaneously capturing the full mutational architecture of the disease. In the landmark study by Manier et al., CTCs were first enriched from peripheral blood by CD138-based immunomagnetic selection, followed by whole-exome sequencing performed at a mean target coverage of 204× across all compartments, including matched cfDNA and tumor biopsies, in a cohort of 107 MM patients [45]. ULP-WGS was additionally applied to derive TF estimates and genome-wide copy number profiles. This dual-platform approach demonstrated concordance in clonal somatic mutations of approximately 99% and in copy number alterations of approximately 81% between liquid biopsy compartments and tumor tissue. Critically, the combined analysis of CTCs and cfDNA enabled cross-validation of mutations, uncovered variants exclusive to either compartment, or extended blood-based tumor profiling to a significantly greater fraction of patients than either analyte alone [82]. Targeted capture NGS panels designed specifically for MM have also demonstrated their capacity to replace, rather than merely complement, FISH in a single integrated assay. Bolli et al. developed a custom 2.3 megabase hybrid capture panel targeting 139 genes alongside the IgH kappa and lambda loci, incorporating a bespoke bioinformatic algorithm to detect chromosomal translocations directly from capture sequencing data without requiring separate FISH probes. In a head-to-head comparison against FISH and SNP microarrays in 154 patients with plasma cell disorders, this panel achieved sensitivity and specificity exceeding 99% for both IgH translocations and copy number alterations, including gain (1q), del (13q), and t (4;14). Importantly, because the panel simultaneously sequences coding mutations across all 139 genes, it identified IgL-MYC translocations and biallelic TP53 inactivation, two high-risk events not captured by standard FISH probe sets, in a subset of patients classified as intermediate-risk by conventional cytogenetics, yielding a hazard ratio for OS of 2.81 (95% CI 1.89–4.17; *p* < 0.0001) in the NGS-defined high-risk group [7]. Complementing these findings, CTC-based targeted NGS by Garcés et al. employed multiparameter NGF cytometry for high-sensitivity CTC isolation, followed by whole-exome and targeted sequencing to simultaneously characterize chromosomal copy number changes, IgH translocations, and somatic mutations across multiple disease compartments in a multiregional, non-invasive manner, capturing clonal heterogeneity from both medullary and extramedullary sites in a single blood draw [62].

For extramedullary disease specifically, comprehensive NGS of 14 tumor samples using a broad targeted panel identified co-occurrence of 1q21 gain/amplification and MAPK pathway mutations in 79% of cases; validation in the large CoMMpass dataset (*n* > 1000 patients) further established that patients harboring mutated KRAS alongside 1q21 gain at diagnosis face a significantly elevated risk of extramedullary progression (HR 2.4; *p* = 0.011), a composite risk profile that individual FISH probes are not designed to detect simultaneously [80]. Collectively, these data demonstrate that whole-exome sequencing and targeted NGS applied to CTCs provide a richer, multi-dimensional genomic readout than FISH, encompassing not only the cytogenetic abnormalities central to current risk stratification but also the somatic mutational landscape, clonal architecture, and spatially distributed subclonal diversity that drive treatment resistance and disease evolution.

## 5. Challenges of the Implementation of Liquid Biopsy in Routine Labs

Despite the promising findings, several limitations and challenges have so far prevented the uptake of liquid biopsies into the clinical setting of MM management. A primary constraint remains the relatively small cohort sizes in published studies, which means larger clinical trials are required to validate the presented findings [83]. Furthermore, standardization of both pre-analytical and analytical workflows is needed, which is particularly critical for progression into clinical settings [26]. Current data suggest that liquid biopsy analysis has complementary utility [34]. However, both cfDNA and CTC-based approaches face distinct technical and biological challenges that limit their integration into routine clinical practice, as listed in Table 3.

Challenges of ctDNA analysis include harmonized collection protocols and a narrow 2–4 h processing window for EDTA blood tubes to prevent leukocyte lysis and genomic DNA contamination [84]. Variants or mutations associated with clonal hematopoiesis may be difficult to distinguish from MM related mutations and hence may not be the ideal marker for monitoring [27]. Systemic factors such as patient age and inflammation affecting cfDNA concentrations, which may influence the sensitivity in detecting ctDNA. Disease factors, including spatial effects and heterogeneity, affect the sensitivity of ctDNA analysis [85].

Several technical and biological limitations also constrain CTC-based approaches. A major challenge is their low abundance in peripheral blood, particularly in low-burden disease states such as MGUS and MRD, which complicates reliable detection and quantification [74]. Furthermore, variability in enrichment and detection methodologies, including differences in flow cytometry sensitivity and microfluidic platforms, contributes to inter-study heterogeneity and limits standardization [86]. In addition, many CTC isolation strategies rely on antibody enrichment, targeting surface markers such as CD138, which introduces a significant selection bias by failing to capture aggressive, CD138-negative subclonal populations that possess higher clonogenic potential and stem-cell-like qualities. This technical hurdle effectively dampens the clinical utility of cellular monitoring, necessitating the shift toward label-free enrichment technologies such as dielectrophoresis or acoustofluidics and enrichment-free detection platforms to ensure a truly representative readout of the entire spatially heterogeneous clonal landscape [45]. The fragility of intact tumor cells also introduces pre-analytical variability, as sample handling and processing delays may affect cell viability and recovery. Additionally, while CTCs provide valuable phenotypic and transcriptomic information, their genomic profiling is often limited by lower DNA yield compared to cfDNA-based approaches [87,88].

The institutional implementation of MRD monitoring requires a careful balance between capital investment and operational scalability. BM-based NGS assays, such as clonoSEQ (approximately $1500–$2000 per test), and ctDNA approaches, including targeted panels and methods such as CAPP-Seq (typically $300–$1000 per sample), require substantial upfront investment in high-throughput sequencing platforms, with instrumentation costs often exceeding $500,000 depending on system configuration [89]. While these approaches offer high analytical sensitivity, their scalability is constrained by multi-step workflows, longer turnaround times, and the need for specialized bioinformatics expertise. Similarly, EuroFlow-based NGF cytometry, although associated with lower capital costs, is limited by relatively high recurring reagent costs (approximately $300–$400 per sample), manual processing requirements, and the need to analyze fresh samples within a 72 h window. In contrast, mass spectrometry-based platforms, such as EXENT, are designed for higher throughput routine monitoring, with reported capacities of up to approximately 100–150 samples per day and increased automation, which may reduce operator dependency and improve workflow efficiency [90]. Some reports suggest that automation may reduce overall laboratory costs; however, estimates of cost savings (up to 50%) are context-dependent and require further validation. Overall, while these technologies show promise, their broader clinical adoption will depend on cost-effectiveness analyses, standardization, and integration into existing laboratory infrastructure. From a clinical perspective, the choice of modality will ultimately depend on institutional resources, available infrastructure, and the intended use case, including longitudinal monitoring versus high-sensitivity MRD assessment.

**Table 3:** Technical and biological barriers to liquid biopsy standardization in MM.

Category	Challenges and Limitations
Pre-analytical Variables	• Inconsistent Procedures: Lack of harmonized protocols for collection, transportation, and temporary storage causes deviating performance [33,84]. • Time-Sensitive Processing: EDTA tubes require plasma separation within 2–4 h to prevent leukocyte lysis for cfDNA [91]. • gDNA Contamination: Release of wild-type genomic DNA (gDNA) from lysed blood cells dilutes marginal ctDNA, impacting detection sensitivity [92]. • CTC Stability: Viability declines with delayed processing (>4–6 h) and is influenced by anticoagulant type and storage conditions [8].
Biological Confounders	• Clonal Hematopoiesis (CH): Somatic variants from blood cells, including age-related clonal hematopoiesis (ARCH) and clonal hematopoiesis of indeterminate potential (CHIP), can mimic tumor-derived mutations, acting as a critical confounder that needs paired white blood cell sequencing and mutation filtering strategies to improve specificity [93]. • Low Tumor Fraction: Plasma ctDNA is often present in marginal amounts, especially in early-stage disease or during remission [94]. • Patient Physiology: cfDNA levels are influenced by non-cancerous factors, including age, gender, exercise, inflammation, and infection. Mean cfDNA in a healthy individual is 30 ng/mL, in MM it is 180 ng/mL, and in MRD-positive patients 20.1–25.2 [26]. • Low CTC Abundance: Typically <1–10 cells/mL (often <1 cell/mL in MRD), limiting detection sensitivity [95].
Disease Specificity	• Spatial Heterogeneity: “Patchy” bone marrow involvement; single-site biopsies or localized disease may not be fully represented in circulating cfDNA [10]. • Compartmental Bias: Discordance between blood and bone marrow results near the limit of detection (e.g., 0.01% VAF) reflects different disease compartments [96]. • Extramedullary Disease: Differences in shedding rates between intra-medullary and extra-medullary plasma cells complicate burden assessment [83].
Technique- Specific Limits	• ddPCR: High (10^−4^–10^−5^) in bone marrow but reduced sensitivity in peripheral blood, particularly in MRD. It is labor-intensive and lacks scalability for blood-based monitoring [26]. •ASO-qPCR: Highly sensitive (10^−5^) but limited by the number of single candidate genetic loci and dependent on patient-specific ASO primers [76]. • NGS Comprehensive Profiling: Facilitates coverage of entire gene panels and identification of novel/epigenetic alterations but lacks standardized guidelines for analysis and data interpretation [97]. • Enrichment Bias in CTCs: Different methods (EpCAM-based, size-based, microfluidic) give variable recovery and may miss some CTC populations [98].
Technical & Analytical	• Fragment Size Loss: Shorter cfDNA fragments carrying critical tumor information can be lost if extraction kits are not optimized. Different kits (e.g., Qiagen silica columns vs. Promega magnetic beads) have various “size-selectivities” [98]. • Sensitivity Gaps: Current cfDNA assays show high specificity (~0.91) but often lower sensitivity compared to standard bone marrow-based MRD assays [83]. • Bioinformatic Variance: Lack of consensus on pipelines for filtering CH variants and interpreting genome-wide data (NGS) [33]. • Low DNA Input for CTCs: Single/few-cell WGA introduces amplification bias, reducing sensitivity for low-VAF mutations in CTCs vs. cfDNA [45].
Clinical & Economic	• Infrastructure Costs: Implementation of standardized NGS and exosome analysis requires high capital investment and specialized equipment [99]. • Validation Deficit: Most cfDNA panels remain in early validation or “proof-of-concept” stages with limited large-cohort prospective data. • Regulatory Timelines: Complex biological products face long pathways (estimated 8–15 years) from discovery to market authorization [100].

MM: multiple myeloma; cfDNA: circulating cell-free DNA; gDNA: genomic DNA; CH: clonal hematopoiesis; ARCH: age-related clonal hematopoiesis; CHIP: clonal hematopoiesis of indeterminate potential; MRD: minimal residual disease; NGS: next-generation sequencing; ddPCR: droplet digital polymerase chain reaction; ASO-qPCR: allele-specific oligonucleotide quantitative polymerase chain reaction; DNA: deoxyribonucleic acid; VAF: varying allele frequency.

## 6. Minimal Residual Disease (MRD)

MRD monitoring in MM is established as a surrogate predictor of PFS and OS. In April 2024, the FDA’s Oncologic Drugs Advisory Committee formally assessed and supported the use of MRD-negative complete response as an early endpoint reasonably likely to predict clinical benefit, supporting its use for accelerated drug approval [20,101]. This endorsement was grounded in comprehensive meta-analyses confirming that achieving MRD negativity at a sensitivity of 10^−5^ by the 12-month mark consistently predicts long-term clinical benefit, with the aim of providing faster access to novel therapies [101]. The 2025 IMWG deep MRD criteria further extended this threshold to 10^−6^, defining a new category that additionally requires the absence of CTCs and no detectable M-protein by mass spectrometry, representing one clonal plasma cell per million total nucleated cells [102]. Against this regulatory and guideline backdrop, there is intense interest in whether peripheral blood-based liquid biopsy can provide MRD information that complements, extends, or in some settings surpasses conventional BM assessment [87].

### 6.1. ctDNA-Based MRD: Methodologies and Evidence

The performance of ctDNA for MRD assessment in MM is critically dependent on the analytical approach employed. Early studies using immunoglobulin gene rearrangements alone produced suboptimal results. Mazzotti et al. demonstrated that when MRD detection uses deep sequencing of IgH V(D)J rearrangements, there is only 49% concordance between paired plasma and BM samples, with ctDNA undetectable in 69% of patients who were confirmed BM MRD positive and a negative predictive value of just 36% [77]. Similarly, Oberle et al. reported a 30% discordance between plasma V(D)J NGS and marrow results, concluding that plasma analysis alone could not replace BM assessment [103]. Using ASO-qPCR targeting patient-specific IgH V(D)J junctional regions, ctDNA analysis demonstrated a sensitivity of 66.7% (22.3–95.7%) and a specificity of 83.3% (35.9–99.6%) when benchmarked against BM MRD detected by multiparameter flow cytometry [103,104]. Consistent with these findings, a meta-analysis of early ctDNA MRD studies in MM, ctDNA IgH gene rearrangement assays, demonstrated high specificity but modest sensitivity (0.58) [83]. The limited and variable sensitivity of IgH VDJ-based approaches is likely attributable to somatic hypermutation affecting primer-binding sites, inadequate sequencing depth, reliance on a single molecular target, and dilutional effects in a background of cfDNA.

In the post-transplant setting, tumor-informed sequencing approaches provide the strongest evidence for ctDNA-based relapse prediction. In a retrospective study of 80 plasma samples from 28 ASCT patients, ctDNA positivity at 3 months post-transplant was strongly predictive of subsequent relapse: 14 of 15 ctDNA-positive patients relapsed on follow-up, with a median PFS of 31 months versus 84 months in ctDNA-negative patients (HR 5.6; 95% CI 1.8–17; *p* = 0.0003) [105]. However, the wide confidence interval warrants caution. Critically, the positive predictive value of ctDNA (93.3%) significantly exceeded that of multiparameter flow cytometry of the BM (68.4%), and multiparameter flow cytometry-based MRD status showed no significant association with PFS (HR 1.2; *p* = 0.73), suggesting that in the post-ASCT setting, tumor-informed blood-based ctDNA may outperform standard BM multiparameter flow cytometry [105].

In the immunotherapy setting, Hosoya et al. utilized CAPP-Seq, an ultra-deep hybrid capture–based targeted sequencing platform designed to interrogate a broad spectrum of coding and non-coding regions, including hypermutated immunoglobulin loci. Target selection was informed by publicly available whole-genome and whole-exome sequencing data. In a limited subset of 16 patients with paired peripheral blood ctDNA-MRD and BM MRD assessments following anti-BCMA CAR T-cell therapy, a strong correlation between ctDNA-based and marrow-based MRD measures was observed (Spearman’s rho = 0.92, *p* = 0.00003) [48]. At day 90 post-infusion, ctDNA-MRD status was significantly associated with time to progression, whereas BM MRD assessed by NGS or NGF cytometry did not demonstrate a statistically significant association with time to progression in this analysis [48]. Notwithstanding these findings, interpretation is constrained by the small number of paired observations and the technical complexity of the assay. While these data support the hypothesis that ctDNA-based MRD assessment may better capture global disease burden and spatial heterogeneity than single-site BM sampling in the post-CAR T-cell setting, prospective validation in larger, uniformly treated cohorts will be required to establish its reproducibility, clinical utility, and incremental value over established marrow-based MRD methodologies [104].

Other established methodologies include ddPCR, which enables absolute quantification of specific targets such as KRAS and BRAF at a sensitivity of 10^−4^ to 10^−5^ and is well suited to cost-effective longitudinal monitoring of patients with known mutations; however, it cannot detect emergent subclones or novel variants [106].

### 6.2. CTC-Based MRD Assessment

The utility of CTCs as a blood-based MRD tool is fundamentally constrained by their extreme rarity in peripheral blood, particularly when disease burden is low. At the depths of sensitivity at which BM NGS or NGF cytometry can reliably detect one clonal plasma cell per 10^6^ total nucleated cells, circulating tumor plasma cells may be present at fewer than one cell per milliliter of blood, which is below the reliable detection threshold of current flow cytometry platforms [107]. To address this critical sensitivity gap, Lasa et al. developed Blood Flow, a novel methodology combining immunomagnetic enrichment of CD138^+^ circulating plasma cells with next-generation flow cytometry, capable of detecting peripheral residual disease below the conventional 2 × 10^−6^ NGF threshold [108]. Utilizing BM MRD status as the reference standard, Blood Flow demonstrated positive and negative predictive values of 95.1% and 76.6%, respectively, with a lower limit of detection of 6 × 10^−8^. Critically, the detection of peripheral residual disease during maintenance or observation was associated with markedly inferior clinical outcomes, with 2-year PFS and OS rates of 0% and 62%, respectively, underscoring the independent prognostic significance of blood-based CTC quantification in the post-treatment setting [108].

Despite this high sensitivity, a significant limitation of such marker-dependent techniques is the potential for antibody masking or epitope competition. In patients receiving anti-CD38 or anti-CD138 directed therapies, the diagnostic antibodies used for magnetic enrichment or flow cytometry gating may be physically blocked, leading to false-negative results or an underestimation of the true CTC burden. This masking effect reinforces the need for either label-free isolation technologies or the use of non-overlapping multi-epitope panels to ensure robust CTC detection across all treatment modalities [67].

### 6.3. Synergistic Application and the Path to Clinical Integration

The IMWG/IMS recommends NGF or NGS of the IgH VDJ region for BM MRD assessment. The minimum sensitivity is 10^−5^, as listed in Table 4. With ongoing prospective studies utilizing advanced technologies, ctDNA and CTC may complement BM MRD, adding prognostic significance [109].

## 7. Mass Spectrometry as a Blood-Based MRD Tool

While ctDNA and CTC-based liquid biopsy approaches provide important genomic insights, complementary proteomic methods are also essential for disease monitoring in MM. The measurement of serum paraproteins remains the cornerstone of response assessment in MM, forming the basis of IMWG response criteria from partial response through to complete response [102]. However, conventional methods of serum protein electrophoresis and immunofixation electrophoresis are limited by insufficient analytical sensitivity at deeper levels of response and are susceptible to interference from therapeutic monoclonal antibodies, most notably daratumumab and isatuximab, which can co-migrate with the patient’s M-protein and confound interpretation [111].

Mass spectrometry addresses these limitations by offering substantially greater sensitivity for M-protein detection, unambiguous discrimination of therapeutic antibody interference, and the practical advantage of requiring only a peripheral blood sample, rendering it well suited to serial, longitudinal monitoring without the burden of BM sampling [112].

Three principal mass spectrometry methodologies have been applied in the MM setting. Matrix-assisted laser desorption/ionization time-of-flight mass spectrometry (MALDI-TOF MS), implemented in platforms such as Mass-Fix and EXENT, is the most widely adopted approach in clinical practice owing to its high throughput and scalability. However, its performance at low M-protein concentrations may be influenced by the polyclonal immunoglobulin background, with improved quantification observed in hypogammaglobulinaemic conditions [113]. Liquid chromatography high-resolution mass spectrometry (LC-HRMS) offers superior resolution and sensitivity through chromatographic separation, enabling improved signal-to-noise ratio and detection sensitivity approaching or exceeding BM-based NGS thresholds [114]. Liquid chromatography tandem mass spectrometry (LC-MS/MS), employing a clonotypic peptide bottom-up approach, provides an orthogonal strategy particularly suited to patients with non-secretory or oligosecretory disease. Prospective clinical studies have demonstrated that mass spectrometry-based response assessment carries independent prognostic significance, outperforming conventional electrophoretic methods in predicting PFS and OS across multiple treatment settings [112].

Among these platforms, the MALDI-TOF-based quantitative immunoprecipitation mass spectrometry (QIP-MS) approach has been most extensively evaluated as a blood-based adjunct for MRD assessment in MM. QIP-MS operates on the principle that each M-protein possesses a unique amino acid sequence and therefore a unique mass, enabling its identification, typing, and quantification in peripheral blood with sensitivity exceeding that of conventional serum electrophoresis and immunofixation. The subsequent commercial development of the EXENT platform (The Binding Site, Thermo Fisher Scientific, Birmingham, UK), which employs polyclonal sheep antibodies directed against immunoglobulin heavy and light chains covalently linked to paramagnetic beads, has translated this methodology into a standardized, scalable clinical assay with a limit of detection of 0.0015 g/dL, facilitating its adoption across multiple prospective trial settings [115].

Clinical validation studies support the prognostic value of mass spectrometry-based assessment. Puig et al. conducted a comparative analysis of QIP-MS in serum against NGF cytometry performed on BM aspirates across multiple treatment time points, spanning induction, transplant, consolidation, and maintenance, in the context of the GEM2012MENOS65 and GEM2014MAIN trials, two prospective phase 3 studies of newly diagnosed, transplant-eligible MM patients. In these studies, QIP-MS and BM NGF achieved equivalent prognostic value, a finding that positions serum mass spectrometry as a viable, minimally invasive complement to established BM-based MRD assessment [18]. Extending these observations to a post-transplant maintenance setting, Kubicki et al. evaluated EXENT-based mass spectrometry MRD assessment in 138 patients enrolled in the ATLAS trial, a phase 3 study of post-transplant maintenance in NDMM patients comparing carfilzomib, lenalidomide, and dexamethasone (KRd) versus lenalidomide alone, reporting a 67–70% agreement between peripheral blood mass spectrometry and BM MRD results at the 10^−5^ threshold assessed by either NGS or multiparameter flow cytometry [116].

Critically, patients achieving concurrent MRD negativity in both peripheral blood by mass spectrometry and BM demonstrated superior PFS compared with those negative in only one compartment, reinforcing the concept that multicompartmental MRD assessment captures a more complete picture of disease eradication than single-site evaluation alone [116,117]. Collectively, these data support the integration of mass spectrometry into a multimodal MRD framework alongside BM NGS and NGF and emerging liquid biopsy approaches, though prospective validation in larger cohorts, optimal timing of assessment, and standardization of mass spectrometry platforms across centers remain essential prerequisites for routine clinical implementation.

## 8. Conclusions

The clinical management of multiple myeloma is evolving in parallel with the increasing effectiveness of contemporary therapies and the growing understanding of the disease’s genomic complexity and spatial heterogeneity. As quadruplet induction regimens, immunotherapy, and CAR T-cell therapies drive unprecedented depths of response, sustained MRD negativity has emerged as a key treatment endpoint and surrogate marker of response. However, progression-free survival and overall survival remain the definitive measures of clinical benefit. In this context, liquid biopsy represents a promising adjunct to conventional disease monitoring, with the potential to provide accessible, repeatable, and minimally invasive assessment that may overcome the fundamental limitation of single-site bone marrow sampling in a spatially heterogeneous malignancy. Through the integrated analysis of ctDNA and CTCs, liquid biopsy may provide complementary, non-redundant insights into clonal architecture, tumor evolution, and treatment resistance mechanisms. Multiple studies have demonstrated the diagnostic, prognostic, and therapeutic significance of these circulating analytes across the disease spectrum, from precursor states to relapsed and refractory disease. However, prospective interventional studies demonstrating improved patient outcomes with ctDNA or CTC-guided strategies are currently lacking. Nevertheless, the clinical translation of liquid biopsy into routine MM management will depend on conducting large, prospective validation studies in the setting of modern therapy, alongside the standardization of pre-analytical workflows, analytical platforms, and regulatory frameworks to ensure reproducibility and clinical utility.

## Figures and Tables

**Table 2:** Comparison of Key Features Between CTCs and cfDNA.

Feature	Circulating Tumour Cells (CTCs)	Circulating Tumor DNA (ctDNA)
Biomarker	• Intact, viable tumor cells from peripheral blood [76]. • Help in determining biology and function	• Tumor-derived DNA fragments in plasma (ctDNA), diluted by a background of normal cell-free DNA (cfDNA) [77]. • High-sensitivity genomic mutation tracking
Key Methodologies	• Next-Generation Flow (NGF) cytometry enumeration and immunophenotypic characterization. • Downstream analysis via WES, scRNA-seq, or targeted NGS [78].	• Targeted NGS, ultrasensitive deep sequencing • ddPCR and ASO-qPCR for tracking known mutations and Ig rearrangements [48,79].
Genomic Profiling	• High concordance (70–90%) between mutations detected in CTCs and bone marrow (BM) samples. • Captures greater spatial heterogeneity, which may represent clones from multiple tumor sites [62]. • The transcriptomic profile shows an “egress signature” enriched for hypoxia and epithelial–mesenchymal transition pathways [64].	• High concordance (up to 96%) between ctDNA-derived mutations and clonal BM mutations. • Frequently detects “plasma-only” mutations missed by BM biopsy, confirming spatial heterogeneity. • More sensitive for mutation detection than sorted BM cells in certain contexts [36,37].
Clinical & Prognostic Value	• Enumeration is a powerful, independent prognostic factor for Progression-Free Survival (PFS) & Overall Survival (OS). • High counts (e.g., ≥2% by NGF in some studies) define an ultra-high-risk patient group with outcomes like plasma cell leukemia [13,80].	• Higher levels are associated with worse PFS & OS. • Specific mutations (TP53, KRAS) in ctDNA are stronger prognostic predictors than their BMPC counterparts in RRMM. • Enables noninvasive longitudinal monitoring, which may anticipate clinical relapse earlier than serum markers [39,41,71].
Major Limitations	• Low prevalence makes isolation difficult. especially in low-burden states like MGUS or MRD. • Lack of standardized enrichment and detection methods creates variability across studies [25,81].	• Dilution by normal cfDNA requires highly sensitive assays to avoid false negatives. • Clonal hematopoiesis (CH) mutations can confound interpretation. • Variable sensitivity for MRD is reported, with some studies showing high false-negative rates [37,41,48].

NGF: next-generation flow cytometry; WES: whole-exome sequencing; scRNA-seq: single-cell RNA sequencing; NGS: next-generation sequencing; ddPCR: droplet digital polymerase chain reaction; ASO-qPCR: allele-specific oligonucleotide quantitative polymerase chain reaction; BMPC: bone marrow plasma cell; PFS: progression-free survival; OS: overall survival; RRMM: relapsed/refractory multiple myeloma; MGUS: monoclonal gammopathy of undetermined significance; MRD: minimal residual disease; CH: clonal hematopoiesis.

**Table 4:** Evolution of liquid biopsy methodologies for MRD.

Methodology	Key Findings/Outcomes	Major Limitations	References
Tumor-Informed WGS: Whole-genome sequencing of cfDNA guided by baseline mutations (~2500).	Achieved sensitivity of 0.011% VAF, or 10^−4^, in tumor-informed settings and predicted relapse with a median lead time of 12.6 months in BM-informed cfDNA MRD and 19 months in plasma cfDNA.	Requires a baseline bone marrow biopsy to “inform” the personalized assay; high bioinformatics cost.	[94]
Targeted Ultra-Deep NGS (CAPP-Seq): Hybrid capture panel incorporating coding, non-coding, and immunoglobulin loci for ctDNA profiling	Inclusion of a large number of mutations (median ~83 SNVs per case) significantly improves MRD detection sensitivity and enables tracking of disease burden, treatment response, and early relapse detection in MM.	Sensitivity depends on the number of detectable mutations, with limited performance in low-tumor-burden cases; it requires sufficient baseline mutation identification.	[48]
Low-Pass WGS (LPWGS): Broad, shallow sequencing to detect CNAs.	Found a ≥10% tumor fraction threshold predicted poor outcomes and identified subclonal evolution in RRMM.	Low sensitivity of 10^−1^ to 10^−2^; insufficient to detect deep MRD (≤10^−5^) in newly diagnosed patients.	[110]
IgH-focused NGS: Deep sequencing of the immunoglobulin heavy chain (IgH) locus.	Found no correlation between blood and BM for MRD when tracking only the IgH locus.	Established that tracking a single locus in blood may be insufficient for high-sensitivity MRD monitoring.	[77]
V(D)J NGS: Sensitivity 10^−5^ to 10^−6^ Analysis of clonotypic rearrangements in cfDNA vs. leukocyte DNA.	cfDNA levels correlated better with treatment response than M-protein due to its shorter half-life.	Reported 30% discordance between plasma V(D)J NGS and bone marrow, indicating that plasma alone was not sufficient to replace BM assessment.	[103]

MRD: minimal residual disease; WGS: whole-genome sequencing; NGS: next-generation sequencing; LPWGS: low-pass whole-genome sequencing; RRMM: relapsed/refractory multiple myeloma; IgH: immunoglobulin heavy chain; V(D)J: variable (diversity), joining gene segments.

## Data Availability

No new data were created or analyzed in this study. Data sharing is not applicable to this article.

## Abbreviations

The following abbreviations are used in this manuscript:

MM	Multiple Myeloma
MGUS	Monoclonal Gammopathy of Undetermined Significance
SMM	Smoldering Multiple Myeloma
RRMM	Relapsed/Refractory Multiple Myeloma
NDMM	Newly Diagnosed Multiple Myeloma
BM	Bone Marrow
BMPCs	Bone Marrow Plasma Cells
PCs	Plasma Cells
cfDNA	Cell-Free DNA
ctDNA	Circulating Tumor DNA
CTCs	Circulating Tumor Cells
EVs	Extracellular Vesicles
TEPs	Tumor-Educated Platelets
MRD	Minimal Residual Disease
PFS	Progression-Free Survival
OS	Overall Survival
LDH	Lactate Dehydrogenase
NGS	Next-Generation Sequencing
WGS	Whole-Genome Sequencing
ULP-WGS	Ultra-Low-Pass Whole-Genome Sequencing
LP-WGS	Low-Pass Whole-Genome Sequencing
WES	Whole-Exome Sequencing
scRNA-seq	Single-Cell RNA Sequencing
ddPCR	Droplet Digital Polymerase Chain Reaction
ASO-qPCR	Allele-Specific Oligonucleotide Quantitative Polymerase Chain Reaction
CAPP-seq	Cancer Personalized Profiling by Deep Sequencing
LB-Seq	Liquid Biopsy Sequencing
CAN	Copy Number Alteration
SNV	Single-Nucleotide Variation
VAF	Variant Allele Frequency
FISH	Fluorescence In Situ Hybridization
PET/CT	Positron Emission Tomography/Computed Tomography
MRI	Magnetic Resonance Imaging
BCMA	B-Cell Maturation Antigen
CAR T-cell	Chimeric Antigen Receptor T-cell
CH	Clonal Hematopoiesis
CHIP	Clonal Hematopoiesis of Indeterminate Potential
IMWG	International Myeloma Working Group
R-ISS	Revised International Staging System
IgH	Immunoglobulin Heavy Chain
FLC	Free Light Chain
EXENT	Commercial MALDI-TOF-based QIP-MS platform
LC-HRMS	Liquid Chromatography High-Resolution Mass Spectrometry
LC-MS/MS	Liquid Chromatography Tandem Mass Spectrometry
MALDI-TOF MS	Matrix-Assisted Laser Desorption/Ionization Time-of-Flight Mass Spectrometry
